# The Influence of Obesity on Small Bowel Capsule Endoscopy

**DOI:** 10.1155/2022/6396651

**Published:** 2022-05-09

**Authors:** Teppei Omori, Yu Sasaki, Miki Koroku, Harutaka Kambayashi, Shun Murasugi, Maria Yonezawa, Shinichi Nakamura, Katsutoshi Tokushige

**Affiliations:** Institute of Gastroenterology, Tokyo Women's Medical University, 8-1 Kawada-cho, Shinjuku-ku, Tokyo 162-8666, Japan

## Abstract

**Objective:**

Intestinal motility may be different in obese and nonobese patients, but this has not been determined. Here, we sought to evaluate the effect of obesity on small bowel capsule endoscopy (SBCE). *Patients and Methods*. We retrospectively analyzed the cases of the 340 patients who underwent SBCE for small intestinal disease (excluding cases of unobservable total small bowel, small bowel stenosis, and bowel resection) at our hospital during the period January 2014 to December 2020 to extract patient background factors and the bowel transit times of SBCE according to the presence/absence of obesity (defined as a body mass index (BMI) ≥ 25 kg/m^2^).

**Results:**

The obese group was 54 patients (nonobese, *n* = 286). The small bowel transit time (SBTT) was significantly shorter in the obese patients compared to the nonobese patients (*p* = 0.0026), and when we divided the patients by their short/long SBTTs using 216.5 min as the cutoff, we observed significant between-group differences in the patients' age (≥60 years) and in the patients' hospitalization status at the time of the SBCE examination. A multivariate analysis revealed that hospitalized status at the examination is a factor contributing significantly to a long SBTT (OR 0.25, 95% CI: 0.15–0.42, *p* < 0.0001). An analysis using the outpatient/inpatient conditions showed that obesity was an independent factor in the inpatient status at the SBCE examination with a significant short SBTT (OR 2.91, 95% CI: 1.06–7.97, *p* = 0.0380). Constipation at the examination was also a factor contributing to a long SBTT (OR 0.26, 95% CI: 0.07–0.99, *p* = 0.0493).

**Conclusion:**

The SBTT of the SBCE was significantly shorter in the obese patients. This tendency was especially evident in the hospitalized state.

## 1. Objective

Humans obtain energy by absorbing nutrients through the digestive tract. An excessive intake of nutrients can cause obesity, and it has been suggested that the efficiency of intestinal nutrient absorption and motility differs between obese and nonobese individuals [1–4], although this has not been established [5]. We conducted the present study to determine whether the gastrointestinal motility of obese subjects differs from that of nonobese subjects by retrospectively analyzing the subjects' small bowel capsule endoscopy (SBCE) results.

## 2. Patients and Methods

### 2.1. Patients

We analyzed the cases of the patients who attended Tokyo Women's Medical University and underwent SBCE between January 2014 and December 2020. Obesity was defined as a body mass index (BMI) ≥ 25 kg/m^2^. The study inclusion criteria were age ≥ 18 years and requiring a small bowel examination by SBCE. The following patients were excluded: those for whom all small bowel observations could not be recorded because the examination was mistakenly terminated before reaching the colon; those with small bowel stenosis that prevented the passage of the PillCam™ patency capsule (PPC) (Medtronic, Minneapolis, MN, USA); patients with severe stenosis on images recorded by SBCE; and patients with a history of bowel resection.

### 2.2. Methods

We divided the patients into obese and nonobese groups by their BMIs and compared the groups' patient background factors and intestinal transit time of SBCE. The mean small bowel transit time (SBTT) of the patient's SBCE was used to classify the patients into the short SBTT group and the long SBTT group, and we analyzed the factors contributing to the faster SBTT.

### 2.3. Small Bowel Capsule Endoscopy

For each SBCE, the patient was instructed to fast from 21 : 00 on the day before his or her SBCE examination. At 8 : 00 on the following morning, mosapride (15 mg) was administered orally as a pretreatment drug. An intestinal cleansing procedure was considered unnecessary for the SBCE. The start of the SBCE procedure was 9 : 00. Drinking water was provided 2 h after, and a meal was allowed 4 h after the SBCE was swallowed. Among the patients whose 12 h fasting state was confirmed, some patients underwent an urgent SBCE examination. The excretion of the capsule endoscope was confirmed visually after the completion of the examination.

Intestinal patency was confirmed in cases of suspected stenosis by a PCC before the SBCE procedure. The PPC is the same size as the SBCE (26 mm long, 11 mm dia); it is made of lactose with 10% barium sulfate and has a film coating. Intestinal fluid enters through two timer plugs, and the capsule begins to dissolve 33 h after oral administration. The small intestinal patency can be assessed by the excretion of the PPC out of the body or its arrival in the colon before a change in the PPC's shape occurs. The SBCE was performed by practitioners with experience conducting >1,000 SBCEs. The capsule endoscopy was the PillCam™ SB2 or SB3 (Medtronic).

The transit times through the esophagus, stomach, and small intestine were determined by marking with the reading software. The time from the esophageal inlet to the gastric hilum was defined as the esophageal transit time (ETT), the time from the gastric hilum to the duodenal bulb as the gastric transit time (GTT), and the time from the duodenal bulb to the cecum as the small bowel transit time (SBTT).

### 2.4. Ethical Considerations

All patients fulfilled the eligibility criteria for SBCE. The indications for an SBCE examination, its risks, and countermeasures against potential complications were carefully explained to each patient, and written informed consent was obtained. The study protocol was reviewed and approved by the Human Ethics Review Committee of Tokyo Women's Medical University (2021-0003).

### 2.5. Statistical Analyses

All data are expressed as the median (interquartile range (IQR)). Wilcoxon's test was used in a univariate analysis of background factors. In the multivariate analysis, odds ratios (ORs) were calculated by a logistic regression analysis. Point estimates and interval estimates for all descriptive data are presented as the mean or proportion, together with the standard deviation or 95% confidence interval (CI). Probability (*p*) values < 0.05 were considered significant. JMP statistical analysis software (ver. 11; SAS, Cary, NC) was used in all analyses.

## 3. Results

### 3.1. Background Factors

The total number of patients who underwent a targeted SBCE examination during the study period was 340; their characteristics are summarized in Table 1. The median (IQR) age was 49 (31–69) years; 157 patients (46.2%) were female and 183 (53.8%) were male. Their height was 163 (155.3–170) cm, weight was 56 (48.1–65) kg, and the median BMI of the entire patient series was 21 (18.8–23.8) kg/m^2^.

Fifty-four (15.9%) patients had a BMI ≥ 25 kg/m^2^, and nine (2.6%) had a BMI ≥ 30 kg/m^2^. The reasons for the SBCE examination were obscure gastrointestinal bleeding in 115 patients (33.8%), inflammatory bowel disease (IBD; including Crohn's disease, ulcerative colitis, unclassified IBD, and intestinal Behcet's disease) in 93 patients (27.4%), suspected inflammatory bowel disease in 48 patients (14.1%), tumor in 28 patients (8.2%), anemia in 18 patients (5.3%), and “others” in 38 patients (11.2%). Complications included hypertension in 101 patients (29.7%), heart disease in 63 (18.5%), diabetes in 45 (13.2%), liver cirrhosis in 17 (5%), hemodialysis in 14 (4.1%), irritable bowel syndrome with diarrhea in 16 (4.7%), constipation in 36 (10.6%), and Crohn's disease in 58 (17.1%). At the time of their SBCEs, 136 patients (40%) were hospitalized (Table 1).

### 3.2. SBCE Intestinal Transit Times

Among all patients, the ETT was 3 (2–8) sec, the GTT was 14 (8–32) min, and the SBTT was 216.5 (155–309) min (Table 1).

### 3.3. Background Factors and the Intestinal Transit Times in the Obese and Nonobese Patients

We compared background factors and complications in the obese (O) group (*n* = 54) and the nonobese (NO) group (*n* = 286), and we found no significant differences in background factors or complications between these groups (Table 2). There was no significant between-group difference in the ETT (O group vs. NO group: 3 (2–5) vs. 3 (2–8) sec, *p* = 0.4057) or the GTT (O group vs. NO group: 12.5 (7.8–33.5) vs. 15 (8–32) min, *p* = 0.6968). However, the SBTT of the O group was significantly shorter than that of the NO group: 177 (122.8–242.9) vs. 227 (162.8–320) min, respectively (*p* = 0.0026).

### 3.4. Factors Influencing the SBTT

We grouped the patients into the short and long SBTT groups by the median SBTT of 216.5 min. The proportions of each group were short SBTT with 170 patients (50%) and long SBTT with 170 patients (50%) (Table 3). The ETT (short SBTT vs. long SBTT: 3 (2–6) vs. 3 (2–8) sec, *p* = 0.3633) and GTT (short SBTT vs. long SBTT: 14 (8–33.3) vs. 15 (8–32) min, *p* = 0.8650) were not significantly different between the groups. The rates of capsule endoscopic findings (erosions/ulcer lesions, active bleeding, and tumors) and urgent examinations were also not significantly different between the long and short SBTT groups. Significant between-group differences were observed in the following background factors: age ≥ 60 years, hypertension, and hospitalized status at the time of SBCE examination. The results of the multivariate analysis showed that male gender and hospitalized status at the time of the examination were factors contributing to a long SBTT (OR 0.62, 95% CI: 0.39–0.99, *p* = 0.0432) and (OR 0.25, 95% CI: 0.15–0.42, *p* < 0.0001), respectively (Table 4).

We examined the factors that affect the SBTT using stratification by the patients' hospital status at the time of the SBCE examination. In the setting of inpatient status, the inpatients were significantly older and were more likely to have hypertension, diabetes, liver cirrhosis, cardiac disease, and constipation, and more likely to be undergoing hemodialysis. The inpatients had significantly lower Hb and Alb and significantly higher BUN and Cr. In contrast, the outpatients had a significantly higher percentage of premedication for SBCE, IBS-D, and CD. The GTT and the SBTT were significantly longer in the inpatients compared to the outpatients (Supplementary Table S1).

Among the background factors of the outpatients, there was no significant difference between the long SBTT group and the short SBTT group (Supplementary Table S2). Among the inpatients, obesity (BMI ≥ 25 kg/m^2^) was an independent factor contributing significantly to short SBTT (OR 2.91, 95% CI: 1.06–7.97, *p* = 0.0380). Constipation at the time of the SBCE examination was revealed as a significant factor contributing to a long SBTT (OR 0.26, 95% CI: 0.07–0.99, *p* = 0.0493) (Supplementary Table S3, Table 5).

As an exploratory study, we calculated the BMI value with the largest area under the curve (AUC), using the Youden index and the receiver operating characteristics (ROC) curve obtained with the median SBTT in the total study population: BMI 23.8 kg/m^2^ (AUC 0.55, Supplementary Figure S1). The results of the multivariate analysis showed that a BMI ≥ 23.8 kg/m^2^ was a significant factor for a short SBTT (OR 2.21, 95% CI: 1.27–3.87, *p* = 0.0051). Hospitalized status at the time of SBCE examination was observed to be a significant factor contributing to a long SBTT (OR 0.26, 95% CI: 0.16–0.43, *p* < 0.0001) (Supplementary Tables S4 and S5). A BMI ≥ 23.8 was an independent factor significantly associated with a short SBTT in the hospitalized patients (OR 3.6, 95% CI: 1.46–8.9, *p* = 0.0055) (Supplementary Table S6).

## 4. Discussion

This was a retrospective study based on the use of capsule endoscopy to investigate whether the gastrointestinal motility of obese patients differs from that of nonobese patients, using a BMI ≥ 25 kg/m^2^ as the definition of obesity. The results of our analyses demonstrated that obesity is a significant factor that shortened the small bowel transit time in SBCE. The hydrogen breath test [6], lactulose breath test [7], and wireless SmartPill™ are also used to evaluate the small bowel transit time [8]. In the hydrogen breath test, when ingested indigestible carbohydrates reach the large intestine, *E. coli* rapidly ferment them, resulting in an increase in the amount of detectable hydrogen excreted in the breath. This method measures the cecum transit time from this change, but large individual differences were observed [3]. A method to measure the intestinal transit time based on differences in pH among intestinal segments by using the wireless Smart Pill™ capable of measuring pH was reported in 2016 [8].

However, the data regarding small bowel transit in obesity are not consistent [5]. In vitro studies have shown that small intestinal smooth muscle of obese patients has increased contractility compared to that of nonobese patients, suggesting faster intestinal emptying and more rapid intestinal transit [2]. However, the intestinal transit velocity of 100 mL of water, measured by the lactulose breath test, was slower in obese subjects than in nonobese subjects [7]. Another study reported no significant difference in the transit time of fluids from the oral cavity to the cecum between obese and lean individuals [9]. In the present study, the small bowel transit time was measured by using SBCE, which is an imaging method to confirm duodenal and cecal arrival. The endoscopic device used in the present study for SBCE does not disintegrate like food, and it maintains a constant body shape. For this reason, it is difficult to determine the gastrointestinal motility of a meal in obese individuals based on study results. On the other hand, when SBCE is performed, the availability of observations of the entire small intestine affects the quality of the examination. The identification of factors that affect the small intestinal transit time in SBCE is thus clinically significant.

Factors affecting the SBTT in examinations by SBCE have been reported; the factors associated with a prolonged SBTT include age > 60 years, male gender, the presence of diabetes, postoperative status, intestinal stenosis, hospitalized status at the time of the examination, and decreased performance status [10–14]. In the present study's univariate analysis, hypertension was significant, but in a multivariate analysis, the significance of this factor disappeared. These factors might be associated with age and gender. Factors that were reported to be associated with a shorter SBTT include premedication (mosapride), younger age, healthy status, and Crohn's disease [15, 16]. In a prospective study of the relationship between the patient's physical activity and the completion of total small bowel observation by SBCE, it was observed that an outpatient SBCE examination along with high physical activity was correlated with a shorter bowel transit time but was not significantly associated with the completion of total small bowel observation [4]. Interestingly, the higher the patient's BMI, the more complete the small bowel observation was and the shorter the total bowel transit time was [4].

The involvement of intestinal hormone secretions has been suggested as a reason for the faster small intestinal transit time of SBCE in obese individuals. In Japanese patients with intestinal bacteria, the Shannon diversity index was significantly higher in the lean group compared to the obese group (*p* < 0.01), but the Bacteroidetes/Firmicutes ratio did not differ between the obese and lean groups [17]. Differences between Japanese and Western subjects were also detected. However, bacterial species with anti-inflammatory properties (e.g., *F. prausnitzii*) were reported to be significantly increased in lean individuals [17]. Some enteric bacteria such as *F. prausnitzii* break down dietary fiber—which is difficult to degrade by host enzymes—into short-chain fatty acids such as acetate, propionate, and butyrate. It is known that these short-chain fatty acids stimulate the secretion of glucagon-like peptide-1 (GLP-1) from colon L cells and peptide YY (PYY) from endocrine cells in the ileum and colon [18]. These enteroendocrine hormones act on the feeding center to control insulin secretion, food intake, and intestinal motility [19]. In 14 patients with type 2 diabetes mellitus, gastrointestinal motility was evaluated by capsule endoscopy before and after the administration of liraglutide, a GLP1 receptor agonist, and it was observed that liraglutide suppressed the patients' duodenal and small intestinal motility [20]. This suggests that obesity-induced changes in the intestinal microbiota may have affected intestinal hormone secretions and promoted intestinal peristalsis.

There are some study limitations to address. This was a single-center, retrospective analysis of patients with or suspected of having small bowel disease. About 80% of the patients were premedicated with mosapride citrate. No measurement of intestinal bacteria or blood GLP-1 was conducted. There is a possibility of unadjusted confounding factors. The present findings demonstrate the influence of obesity with regard to the intestinal transit of an endoscopic device used in SBCE; in other words, the results cannot be generalized to dietary gastrointestinal motility. Nevertheless, the identification of factors affecting the small intestinal transit time of SBCE has clinical significance. We observed that the patients' SBCE enabled the accurate determination of their intestinal transit times because the use of SBCE can determine the passage through the intestinal tract based on objective images. However, it should be emphasized that this study excluded patients with intestinal resection and obvious small bowel stenosis, thus excluding patients with prolonged transit times due to organic factors.

The present sample size (*n* = 340) is relatively large compared to other studies. We also performed a stratified analysis of inpatient/outpatient background factors, which were considered as confounders in previous studies. Our results showed that the admitted inpatients were significantly older and more often had comorbidities including constipation. They were also more likely to have anemia and hypoalbuminemia and were less physically active; they showed significantly longer gastric transit times and small bowel transit times. Nevertheless, the small bowel transit time was significantly shorter in the obese patients during the SBCE examination in the hospitalized state (in which the patients' level of physical activity was lower). This may have the effect of decreasing the detectability of small bowel lesions by SBCE. For this reason, a more careful reading of SBCE images is necessary, and premedication to stimulate bowel motility should be avoided.

In conclusion, the small bowel transit time of a capsule endoscope in obese subjects was faster than that in nonobese subjects, and this feature was more pronounced in the hospitalized patients. The mechanism of obesity-induced changes in small intestinal motility deserves further study in conjunction with analyses of intestinal bacteria and enteroendocrine hormones.

## Figures and Tables

**Table 1:** The patients' characteristics (*n* = 340).

Males	183 (53.8)
Age (yrs)	49 (31-69)
Preparation (15 mg mosapride)	283 (83.2)
Height (cm)	163 (155.3-170)
Weight (kg)	56 (48.1-65)
BMI (kg/m^2^)	21 (18.8-23.8)
BMI ≥ 25 kg/m^2^	54 (15.9)
BMI ≥ 30 kg/m^2^	9 (2.6)
Objective of the examination	
Obscure GI bleeding	115 (33.8)
IBD	93 (27.4)
IBD suspected	48 (14.1)
Tumor	28 (8.2)
Anemia	18 (5.3)
Others	38 (11.2)
Comorbidities	
Hypertension	101 (29.7)
Heart disease	63 (18.5)
Diabetes mellitus	45 (13.2)
Liver cirrhosis	17 (5)
Hemodialysis	14 (4.1)
Diarrhea-predominant IBS	16 (4.7)
Crohn's disease	58 (17.1)
Constipation	36 (10.6)
Laboratory data	
Hemoglobin (g/dL)	11.9 (9.5–13.7)
Platelet (×10^44^/*μ*L)	23 (18.3–29.6)
Albumin (g/dL)	3.9 (3.3–4.4)
BUN (mg/dL)	13.3 (10.2–21)
Cr (mg/dL)	0.79 (0.65–0.98)
Intestinal transit time	
ETT (sec)	3 (2–8)
GTT (min)	14 (8–32)
SBTT (min)	216.5 (155–309)
Inpatient examination	136 (40)
Urgent examination	22 (6.5)

The data are median (interquartile range) or number (%) of patients. BMI: body mass index; BUN: blood urea nitrogen; Cr: creatinine; ETT: esophagus transit time; GI: gastrointestinal; GTT: gastric transit time; IBD: inflammatory bowel disease; IBS: irritable bowel syndrome; SBTT: small bowel transit time.

**Table 2:** Background factors and intestinal transit times with and without obesity.

	Obesity (O) (*n* = 54) (%)	Nonobesity (NO) (*n* = 286) (%)	*p* value
Males	30 (55.6)	153 (53.5)	0.8819
Age (yrs)	55.5 (41–63.8)	47 (29.8–69)	0.1947
Preparation (15 mg mosapride)	44 (81.5)	239 (83.6)	0.6935
Height (cm)	163.3 (154.8–170.3)	163 (155.5–169.6)	0.6952
Weight (kg)	73.5 (67–80)	53.6 (41.9–60)	<0.0001
BMI (kg/m^2^)	27 (25.9–28.5)	20.4 (18.4–22.4)	<0.0001
Comorbidities			
Hypertension	18 (33.3)	83 (29)	0.5200
Heart disease	12 (22.2)	51 (17.8)	0.4478
Diabetes mellitus	10 (18.5)	35 (12.2)	0.2712
Liver cirrhosis	5 (9.3)	12 (4.2)	0.1628
Hemodialysis	4 (7.4)	10 (3.5)	0.2511
IBS-D	2 (3.7)	14 (4.9)	1.0000
Crohn's disease	6 (11.1)	52 (18.2)	0.2410
Constipation	6 (11.1)	30 (10.5)	0.8132
Laboratory data			
Hemoglobin (g/dL)	11.8 (9.3–14.2)	12 (9.5–13.7)	0.9464
Platelet (×10^44^/*μ*L)	23.2 (19.2–30.7)	22.9 (18.2–29.2)	0.5971
Albumin (g/dL)	4.1 (3.6–4.4)	3.9 (3.3–4.4)	0.2588
BUN (mg/dL)	15.3 (11.7–32.3)	13.2 (10–19.7)	0.0895
Cr (mg/dL)	0.81 (0.66–1.08)	0.79 (0.65–0.97)	0.4815
Intestinal transit time			
ETT (sec)	3 (2–5)	3 (2–8)	0.4057
GTT (min)	12.5 (7.8–33.5)	15 (8–32)	0.6968
SBTT (min)	177 (122.8–242.9)	227 (162.8–320)	0.0026
SBCE findings			
Erosion, ulcer	19 (35.2)	104 (36.4)	1.0000
Active bleeding	4 (7.4)	24 (8.4)	1.0000
Tumor	2 (3.7)	22 (7.7)	0.3943
Inpatient examination	20 (37)	116 (40.6)	0.6531
Urgent examination	5 (9.3)	17 (5.9)	0.3664

The data are median (interquartile range) or number (%) of patients. BMI: body mass index; BUN: blood urea nitrogen; Cr: creatinine; IBS-D: diarrhea-predominant irritable bowel syndrome; ETT: esophagus transit time; GTT: gastric transit time; SBTT: small bowel transit time.

**Table 3:** Background factors and intestinal transit time grouped by small intestinal transit time.

	Long SBTT (*n* = 170) (%)	Short SBTT (*n* = 170) (%)	*p* value
Males	100 (58.8)	83 (48.8)	0.0816
Age (yrs)	53.5 (32–72)	44.5 (28.8–63.3)	0.0163
Age ≥ 60 yrs	74 (43.5)	50 (29.4)	0.0094
Preparation (15 mg mosapride)	135 (79.4)	148 (87.1)	0.0808
Height (cm)	164 (156.4–170)	161.7 (155–170)	0.3573
Weight (kg)	55.9 (48.2–64.3)	56.4 (48–67)	0.4927
BMI (kg/m^2^)	20.8 (18.7–23.2)	21.4 (18.8–24.2)	0.1323
BMI ≥ 25 kg/m^2^	21 (12.4)	33 (19.4)	0.1020
BMI ≥ 23.8 kg/m^2^	31 (18.2)	52 (30.6)	0.0113
Comorbidities			
Hypertension	61 (35.9)	40 (23.5)	0.0174
Heart disease	39 (22.9)	24 (14.1)	0.0501
Diabetes mellitus	27 (15.9)	18 (10.6)	0.2001
Liver cirrhosis	12 (7.1)	5 (2.9)	0.1334
Hemodialysis	8 (4.7)	6 (3.5)	0.7861
IBS-D	7 (4.1)	9 (5.3)	0.7988
Crohn's disease	29 (17.1)	29 (17.1)	1.0000
Constipation	24 (14.1)	12 (7.1)	0.0513
Intestinal transit times			
ETT (sec)	3 (2–6)	3 (2–8)	0.3633
GTT (min)	14 (8–33.3)	15 (8–32)	0.8650
SBTT (min)	308.5 (254–390.3)	155 (107.8–188.3)	<0.0001
SBCE findings			
Erosion, ulcer	62 (36.5)	61 (35.9)	1.0000
Active bleeding	13 (7.7)	15 (8.8)	0.8440
Tumor	12 (7.1)	12 (7.1)	1.0000
Inpatient examination	96 (56.5)	40 (23.5)	<0.0001
Urgent examination	15 (8.8)	7 (4.1)	0.1211

The data are median (interquartile range) or the number (%) of patients.

**Table 4:** Univariate and multivariate analyses of factors related to short SBTT.

Parameter	Long SBTT (*n* = 170) (%)	Short SBTT (*n* = 170) (%)	Univariate analysis (*p* value)	Multivariate analysis		
				*p* value	OR	95% CI
Males	100 (58.8)	83 (48.8)	0.0816	0.0432	0.62	0.39–0.99
Age ≥ 60 (yrs)	74 (43.5)	50 (29.4)	0.0094			
Preparation (15 mg mosapride)	135 (79.4)	148 (87.1)	0.0808			
BMI ≥ 25 kg/m^2^	21 (12.4)	33 (19.4)	0.1020	0.0826	1.77	0.93–3.35
Hypertension	61 (35.9)	40 (23.5)	0.0174			
Heart disease	39 (22.9)	24 (14.1)	0.0501			
Constipation	24 (14.1)	12 (7.1)	0.0513			
Inpatient examination	96 (56.5)	40 (23.5)	<0.0001	<0.0001	0.25	0.15–0.42

The data are the number (%) of patients.

**Table 5:** Univariate and multivariate analyses of factors related to short SBTT in inpatient.

Parameter	Long SBTT (*n* = 96) (%)	Short SBTT (*n* = 40) (%)	Univariate analysis (*p* value)	Multivariate analysis		
				*p* value	OR	95% CI
Males	56 (58.3)	16 (40)	0.0606			
BMI ≥ 25 kg/m^2^	9 (9.4)	11 (27.5)	0.0144	0.0380	2.91	1.06–7.97
Crohn's disease	14 (14.6)	1 (2.5)	0.0671			
Constipation	20 (20.8)	3 (7.5)	0.0783	0.0493	0.26	0.07–0.99

Data are the number (%) of patients.

## Data Availability

The capsule endoscopic transit time data used to support the findings of this study are available from the corresponding author upon request.

## Abbreviations

BMI: Body mass index
ETT: Esophagus transit time
GLP-1: Glucagon-like peptide-1
GTT: Gastric transit time
PYY: Peptide YY
PPC: PillCam patency capsule
SBCE: Small bowel capsule endoscopy
SBTT: Small bowel transit time.

## Supplementary Materials

Supplementary 1 Supplementary Table S1: background factors and intestinal transit time of the in- and outpatients. Supplementary Table S2: background factors and intestinal transit time grouped by small intestinal transit time in outpatients. Supplementary Table S3: background factors and intestinal transit time grouped by small intestinal transit time in the inpatients. Supplementary Table S4: background factors and intestinal transit time with and without BMI 23.8 kg/m^2^. Supplementary Table S5: univariate and multivariate analyses of factors related to short SBTT with BMI ≥ 23.8 kg/m^2^. Supplementary Table S6: univariate and multivariate analyses of factors related to short SBTT in inpatients with BMI ≥ 23.8 kg/m^2^.

Supplementary 2 Supplementary Figure 1: the BMI value with the largest area under the curve (AUC).
