# Supplementary material for: The Influence of Obesity on Small Bowel Capsule Endoscopy
Source: Gastroenterol Res Pract. 2022 May 9;2022:6396651. doi: 10.1155/2022/6396651 (PMC9112179; doi:10.1155/2022/6396651)
Supplement: Supplementary 1 — Supplementary Table S1: background factors and intestinal transit time of the in- and outpatients. Supplementary Table S2: background factors and intestinal transit time grouped by small intestinal transit time in outpatients. Supplementary Table S3: background factors and intestinal transit time grouped by small intestinal transit time in the inpatients. Supplementary Table S4: background factors and intestinal transit time with and without BMI 23.8 kg/m2. Supplementary Table S5: univariate and multivariate analyses of factors related to short SBTT with BMI ≥ 23.8 kg/m2. Supplementary Table S6: univariate and multivariate analyses of factors related to short SBTT in inpatients with BMI ≥ 23.8 kg/m2. [file 6396651.f1.docx]

**Suppl. Table S1.** Background factors and intestinal transit time of the in- and outpatients

|  | **Inpatients**  **n=136 (%)** | **Outpatients**  **n=204 (%)** | **p-value** |
| --- | --- | --- | --- |
| Males | 72 (52.9) | 111 (54.4) | 0.8247 |
| Age, yrs | 60 [37.3–73] | 42 [29–61] | <0.0001 |
| Preparation, 15 mg mosapride | 99 (72.8) | 184 (90.2) | <0.0001 |
| Height, cm | 162.7 [154–169.7] | 163.8 [156.7–170] | 0.1237 |
| Weight, kg | 55 [48–64] | 57 [48.6–66] | 0.1390 |
| BMI, kg/m^2^ | 20.8 [18.8–23.4] | 21.2 [18.8–23.9] | 0.5165 |
| BMI ≥23.8 kg/m^2^ | 29 (21.3) | 54 (26.5) | 0.3044 |
| BMI ≥25 kg/m^2^ | 20 (14.7) | 34 (16.7) | 0.6531 |
| BMI ≥30 kg/m^2^ | 5 (3.7) | 4 (2) | 0.4925 |
| Comorbidities: |  |  |  |
| Hypertension | 56 (41.2) | 45 (22.1) | 0.0003 |
| Heart disease | 45 (33 | 18 (8.8) | <0.0001 |
| Diabetes mellitus | 26 (19.1) | 19 (9.3) | 0.0136 |
| Liver cirrhosis | 14 (10.3) | 3 (1.5) | 0.0005 |
| Hemodialysis | 12 (8.8) | 2 (1.0) | 0.0005 |
| IBS-D | 2 (1.5) | 14 (6.9) | 0.0332 |
| Crohn’s disease | 15 (11) | 43 (21.1) | 0.0183 |
| Constipation | 23 (16.9) | 13 (6.4) | 0.0035 |
| Laboratory data: |  |  |  |
| Hemoglobin, g/dL | 9.7 [7.7–11.6] | 13 [11.8–14.5] | <0.0001 |
| Platelet, ×10^4^/μL | 22.7 [15.8–32.5] | 23.1 [19.7–28] | 0.4656 |
| Albumin, g/dL | 3.3 [2.9–3.7] | 4.3 [3.9–4.5] | <0.0001 |
| BUN, mg/dL | 18.2 [10.4–33.3] | 12.7 [10.1–15.3] | 0.0002 |
| Cr, mg/dL | 0.92 [0.67–1.59] | 0.76 [0.63–0.88] | <0.0001 |
| Intestinal transit time: |  |  |  |
| ETT, sec [95%CI] | 3 [2–9] | 3 [2–6] | 0.1210 |
| GTT, min [95%CI] | 16 [11–40.8] | 13 [8–26.8] | 0.0044 |
| SBTT, min [95%CI] | 280 [206–385.1] | 190.5 [132.3–248.8] | <0.0001 |
| SBCE findings: |  |  |  |
| Erosion, ulcer | 50 (36.8) | 73 (35.8) | 0.9084 |
| Active bleeding | 19 (14) | 9 (4.4) | 0.0023 |
| Tumor | 8 (5.9) | 16 (7.8) | 0.5260 |
| Urgent examination | 22 (16.2) | 0 (0) | <0.0001 |

The data are median [interquartile range] or number (%) of patients. BMI: body mass index, BUN: blood urea nitrogen, Cr: creatinine, ETT: esophagus transit time, GTT: gastric transit time, IBS-D: diarrhea-predominant irritable bowel syndrome, SBTT: small bowel transit time.

**Suppl. Table S2.** Background factors and intestinal transit time grouped by small intestinal transit time in outpatients

|  | **Long-SBTT**  **n=74 (%)** | **Short-SBTT**  **n=130 (%)** | **p-value** |
| --- | --- | --- | --- |
| Males | 44 (59.5) | 67 (51.5) | 0.3077 |
| Age, yrs | 44.5 [30–66.3] | 39.5 [27–58.3] | 0.01255 |
| Age ≥60 yrs | 26 (35.1) | 29 (22.3) | 0.0510 |
| Preparation, 15 mg mosapride | 66 (89.2) | 118 (90.8) | 0.8075 |
| Height, cm | 164 [158–170] | 163.3 [156.3–170] | 0.5052 |
| Weight, kg | 57 [48.8–67.3] | 57 [48.4–66] | 0.8061 |
| BMI, kg/m^2^ | 21.5 [18.5–23.8] | 21 [18.8–24] | 0.9136 |
| BMI ≥25 kg/m^2^ | 12 (16.2) | 22 (16.9) | 1.0000 |
| BMI ≥23.8 kg/m^2^ | 18 (24.3) | 36 (27.7) | 0.6249 |
| Comorbidities: |  |  |  |
| Hypertension | 18 (24.3) | 27 (20.8) | 0.6000 |
| Heart disease | 7 (9.5) | 11 (8.5) | 0.8023 |
| Diabetes mellitus | 7 (9.5) | 12 (9.2) | 1.0000 |
| Liver cirrhosis | 0 (0) | 3 (2.3) | 0.5550 |
| Hemodialysis | 1 (1.4) | 1 (0.8) | 1.0000 |
| IBS-D | 5 (6.8) | 9 (6.9) | 1.0000 |
| Crohn's disease | 15 (20.3) | 28 (21.5) | 0.8605 |
| Constipation | 4 (5.4) | 9 (6.9) | 0.7728 |
| SBCE findings: |  |  |  |
| Erosion, ulcer | 26 (35.1) | 47 (36.2) | 1.0000 |
| Active bleeding | 1 (1.4) | 8 (6.2) | 0.1600 |
| Tumor | 8 (10.8) | 8 (6.2) | 0.2814 |

The data are median [interquartile range] or number (%) of patients. Abbreviations are explained in the footnote of Suppl. Table S1.

**Suppl. Table S3.** Background factors and intestinal transit time grouped by small intestinal transit time in the inpatients

|  | **Long-SBTT**  **n=96 (%)** | **Short-SBTT**  **n=40 (%)** | **p–value** |
| --- | --- | --- | --- |
| Males | 56 (58.3) | 16 (40) | 0.0606 |
| Age, yrs | 59 [38.3–73] | 61.5 [35.3–73] | 0.7580 |
| Age ≥60 yrs | 48 (50) | 21 (52.5) | 0.8518 |
| Preparation, 15 mg mosapride | 69 (71.9) | 30 (75) | 0.8334 |
| Height, cm | 164 [154.8–169.7] | 158.3 [150–169.4] | 0.1234 |
| Weight, kg | 55 [48.1–62.5] | 54.8 [47–69.2] | 0.5795 |
| BMI, kg/m^2^ | 20.5 [18.7–22.8] | 22 [18.8–25.7] | 0.0446 |
| BMI ≥25 kg/m^2^ | 9 (9.4) | 11 (27.5) | 0.0144 |
| BMI ≥23.8l g/m^2^ | 13 (13.5) | 16 (40) | 0.0011 |
| Comorbidities: |  |  |  |
| Hypertension | 43 (44.8) | 13 (32.5) | 0.2512 |
| Heart disease | 32 (33.3) | 13 (32.5) | 1.0000 |
| Diabetes mellitus | 20 (20.8) | 6 (15) | 0.4834 |
| Liver cirrhosis | 12 (12.5) | 2 (5) | 0.2320 |
| Hemodialysis | 7 (7.3) | 5 (12.5) | 0.3350 |
| IBS-D | 2 (2.1) | 0 (0) | 1.0000 |
| Crohn's disease | 14 (14.6) | 1 (2.5) | 0.0671 |
| Constipation | 20 (20.8) | 3 (7.5) | 0.0783 |
| SBCE findings: |  |  |  |
| Erosion, ulcer | 36 (37.5) | 14 (35) | 0.8468 |
| Active bleeding | 12 (12.5) | 7 (17.5) | 0.4299 |
| Tumor | 4 (4.2) | 4 (10) | 0.2335 |
| Urgent examination | 15 (15.6) | 7 (17.5) | 0.8014 |

The data are median [interquartile range] or number (%) of patients. Abbreviations are explained in the footnote of Suppl. Table S1.

**Suppl. Table S4.** Background factors and intestinal transit time with and without BMI 23.8 kg/m^2^

|  | **BMI ≥23.8 kg/m^2^**  **n=83 (%)** | **BMI <23.8 kg/m^2^**  **n=257 (%)** | **p–value** |
| --- | --- | --- | --- |
| Males | 53 (63.9) | 130 (50.6) | 0.0426 |
| Age, yrs | 56 [42–66] | 45 [28–69] | 0.0246 |
| Preparation, 15 mg mosapride | 67 (80.7) | 216 (84.1) | 0.5007 |
| Height, cm | 165 [155–171.5] | 163 [155.4–169.2] | 0.1968 |
| Weight, kg | 71.6 [65–78.4] | 52 [46–59] | <0.0001 |
| BMI, kg/m^2^ | 25.9 [24.5–27.4] | 20.1 [18.2–21.6] | <0.0001 |
| Comorbidities: |  |  |  |
| Hypertension | 31 (37.4) | 70 (27.2) | 0.0971 |
| Heart disease | 18 (21.7) | 45 (17.5) | 0.4179 |
| Diabetes mellitus | 18 (21.7) | 27 (10.5) | 0.0144 |
| Liver cirrhosis | 6 (7.2) | 11 (4.3) | 0.3826 |
| Hemodialysis | 5 (6) | 9 (3.5) | 0.3426 |
| IBS-D | 3 (3.6) | 13 (5.1) | 0.7696 |
| Crohn’s disease | 12 (14.5) | 46 (17.9) | 0.5075 |
| Constipation | 10 (12.1) | 26 (10.1) | 0.6817 |
| Laboratory data: |  |  |  |
| Hemoglobin, g/dL | 12.6 [9.5–14.3] | 11.8 [9.4–13.5] | 0.1944 |
| Platelet, ×10^4^/μL | 22.6 [18.5–28.4] | 23 [18.2–30.4] | 0.7853 |
| Albumin, g/dL | 4.2 [3.7–4.4] | 3.9 [3.3–4.4] | 0.1075 |
| BUN, mg/dL | 15.5 [11.5–23.3] | 13.1 [10–19.6] | 0.0434 |
| Cr, mg/dL | 0.82 [0.67–1.0] | 0.79 [0.63–0.97] | 0.2590 |
| Intestinal transit time: |  |  |  |
| ETT, sec | 3 [2–5] | 3 [2–8] | 0.4227 |
| GTT, min | 16 [8–36] | 14 [8–31] | 0.3739 |
| SBTT, min | 189 [139–245] | 232 [163.5–321.5] | 0.0012 |
| SBCE findings: |  |  |  |
| Erosion, Ulcer | 26 (31.3) | 97 (37.7) | 0.3578 |
| Active bleeding | 7 (8.4) | 21 (8.2) | 1.0000 |
| Tumor | 8 (9.6) | 16 (6.2) | 0.3246 |
| Inpatient examination | 29 (34.9) | 107 (41.6) | 0.3044 |
| Urgent examination | 8 (9.6) | 14 (5.5) | 0.2001 |

The data are median [interquartile range] or number (%) of patients. BMI: body mass index, BUN: blood urea nitrogen, Cr: creatinine, IBS-D: diarrhea-predominant irritable bowel syndrome, ETT: esophagus transit time, GTT: gastric transit time, SBTT: small bowel transit time.

**Suppl. Table S5.** Univariate and multivariate analysis of factors related to Short-SBTT with BMI ≥23.8 kg/m^2^

| **Parameter** | **Long-SBTT**  **n=170 (%)** | **Short-SBTT**  **n=170 (%)** | **Univariate analysis** |  | **Multivariate**  **analysis** | | |
| --- | --- | --- | --- | --- | --- | --- | --- |
|  |  |  | **p-value** |  | **p-value** | **OR** | **95%CI** |
| Males | 100 (58.8) | 83 (48.8) | 0.0816 |  | 0.0217 | 0.57 | 0.36–0.92 |
| Age ≥60, yrs | 74 (43.5) | 50 (29.4) | 0.0094 |  |  |  |  |
| Preparation, 15 mg mosapride | 135 (79.4) | 148 (87.1) | 0.0808 |  |  |  |  |
| BMI ≥23.8 kg/m^2^ | 31 (18.2) | 52 (30.6) | 0.0113 |  | 0.0051 | 2.21 | 1.27–3.87 |
| Hypertension | 61 (35.9) | 40 (23.5) | 0.0174 |  |  |  |  |
| Heart disease | 39 (22.9) | 24 (14.1) | 0.0501 |  |  |  |  |
| Constipation | 24 (14.1) | 12 (7.1) | 0.0513 |  |  |  |  |
| Inpatient examination | 96 (56.5) | 40 (23.5) | <0.0001 |  | <0.0001 | 0.26 | 0.16–0.43 |

Data are number (%) of patients.

**Suppl. Table S6.** Univariate and multivariate analysis of factors related to Short-SBTT in inpatients with BMI ≥23.8 kg/m^2^

| **Parameter** | **Long-SBTT**  **n=96 (%)** | **Short-SBTT**  **n=40 (%)** | **Univariate analysis** |  | **Multivariate**  **analysis** | | |
| --- | --- | --- | --- | --- | --- | --- | --- |
|  |  |  | **p-value** |  | **p- value** | **OR** | **95%CI** |
| Males | 56 (58.3) | 16 (40) | 0.0606 |  |  |  |  |
| BMI ≥23.8 kg/m^2^ | 13 (13.5) | 16 (40) | 0.0011 |  | 0.0055 | 3.6 | 1.46–8.9 |
| Crohn's disease | 14 (14.6) | 1 (2.5) | 0.0671 |  |  |  |  |
| Constipation | 20 (20.8) | 3 (7.5) | 0.0783 |  | 0.0605 | 0.28 | 0.07–1.06 |

Data are number (%) of patients.
