# Supplementary figures and images for: The Influence of Obesity on Small Bowel Capsule Endoscopy
Source: Gastroenterol Res Pract. 2022 May 9;2022:6396651. doi: 10.1155/2022/6396651 (PMC9112179; doi:10.1155/2022/6396651)

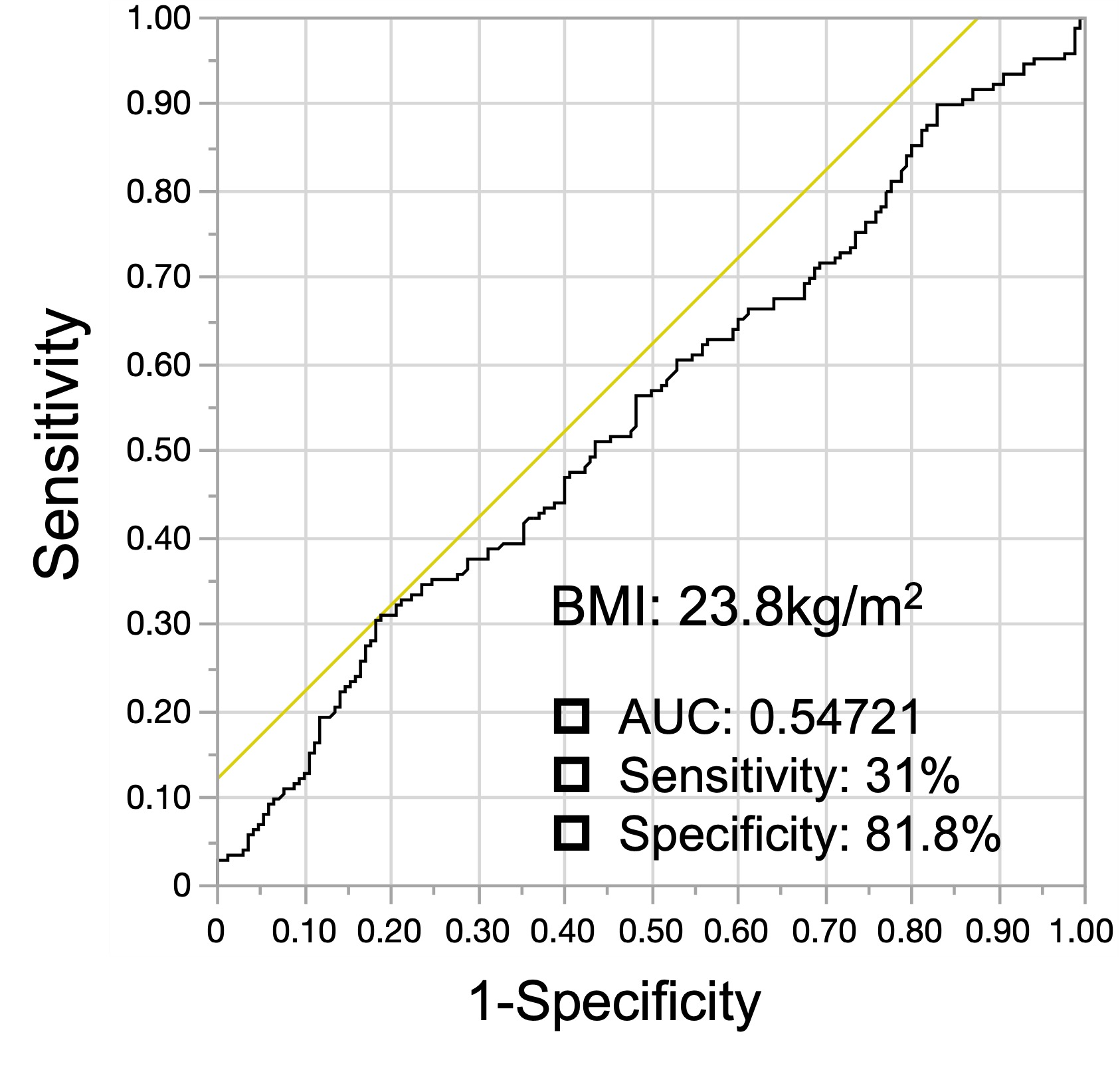

Supplement: Supplementary 2 — Supplementary Figure 1: the BMI value with the largest area under the curve (AUC). [file 6396651.f2.jpg]
